# Diagnosis and management of invasive fungal diseases in non-neutropenic ICU patients, with focus on candidiasis and aspergillosis: a comprehensive review

**DOI:** 10.3389/fcimb.2024.1256158

**Published:** 2024-03-05

**Authors:** Afzal Azim, Armin Ahmed

**Affiliations:** ^1^ Department of Critical Care Medicine, Sanjay Gandhi Post Graduate Institute of Medical Sciences (SGPGI), Lucknow, India; ^2^ Department of Critical Care Medicine, King George’s Medical University, Lucknow, India

**Keywords:** invasive fungal disease, invasive fungal infection, invasive candidiasis, invasive aspergillosis, antifungal drugs

## Abstract

Invasive fungal diseases pose a significant threat to non-neutropenic ICU patients, with *Candida* and *Aspergillus* infections being the most common. However, diagnosing these infections in the ICU population remains challenging due to overlapping clinical features, poor sensitivity of blood cultures, and invasive sampling requirements. The classical host criteria for defining invasive fungal disease do not fully apply to ICU patients, leading to missed or delayed diagnoses. Recent advancements have improved our understanding of invasive fungal diseases, leading to revised definitions and diagnostic criteria. However, the diagnostic difficulties in ICU patients remain unresolved, highlighting the need for further research and evidence generation. Invasive candidiasis is the most prevalent form of invasive fungal disease in non-neutropenic ICU patients, presenting as candidemia and deep-seated candidiasis. Diagnosis relies on positive blood cultures or histopathology, while non-culture-based techniques such as beta-D-glucan assay and PCR-based tests show promise. Invasive aspergillosis predominantly manifests as invasive pulmonary aspergillosis in ICU patients, often associated with comorbidities and respiratory deterioration in viral pneumonia. Diagnosis remains challenging due to poor sensitivity of blood cultures and difficulties in performing lung biopsies. Various diagnostic criteria have been proposed, including mycological evidence, clinical/radiological factors and expanded list of host factors. Non-culture-based techniques such as galactomannan assay and PCR-based tests can aid in diagnosis. Antifungal management involves tailored therapy based on guidelines and individual patient factors. The complexity of diagnosing and managing invasive fungal diseases in ICU patients underscore the importance of ongoing research and the need for updated diagnostic criteria and treatment approaches. Invasive fungal disease, Invasive fungal infection, Invasive candidiasis, Invasive aspergillosis, Antifungal drugs.

## Background

**Figure 1:**
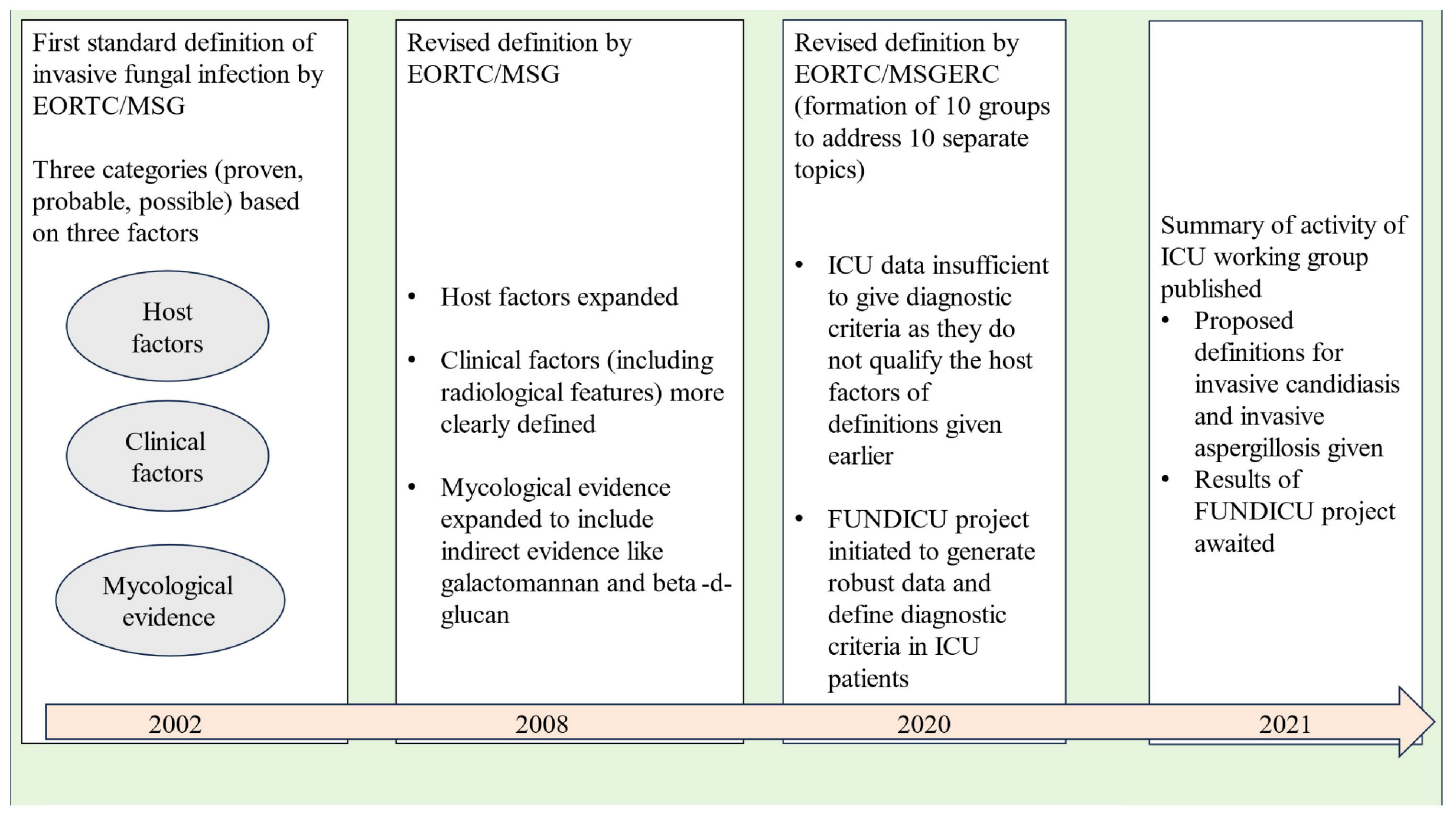
Evolution of our understanding of invasive fungal disease in ICU.

Invasive fungal infections account for 3.8 million deaths annually across the globe, with about 68% (range 35-90%) of these fatalities directly attributed to the fungal infections themselves (Denning, 2024). With recent advancements in healthcare facilities, fungal infections are becoming increasingly important among ICU patients as the number of patients surviving polymorbidity, organ transplantation, and those requiring different forms of life support are increasing (Pappas et al., 2018). Diagnosis of invasive fungal disease (IFD) in the ICU population remains a challenging task due to various issues like the overlap of clinical features with bacterial infections, poor sensitivity and delayed time to positivity of blood cultures, invasive nature of sampling required for diagnosis, etc. However, more importantly, the ICU patient cohort does not fit into “the classical host” described for defining IFD in the scientific literature (Bassetti et al., 2017). ICU patients may show variable grades of alteration in immune response ranging from functional defects to alteration in the subset of cells belonging to innate or adaptive immunity (Serrano et al., 2023). These defects may predispose ICU patients to different forms of invasive fungal infections, but they still do not qualify the classical host criteria of immunocompromised patients. As a result, the diagnosis of IFDs is frequently missed or delayed in the ICU population. In a study from the United States, it has been estimated that the in-hospital mortality or case fatality ratios due to invasive fungal diseases (IFDs) could be five times higher than reported, since death certificates often underestimate the number of deaths caused by fungal infections (Benedict et al., 2022). Before we dig into the intricacies of diagnosing IFDs in the ICU population, it is important to know how our understanding of fungal infections evolved over the past two decades (see **Figure 1**).

In the late nineties, the morbidity and mortality caused by opportunistic fungal infections in immunocompromised hosts was becoming evident to the scientific community. In, 2002, the members of the European Organization for Research and Treatment of Cancer (EORTC)/Invasive Fungal Infections Cooperative Group (IFICG), the National Institute of Allergy and Infectious Diseases (NIAID), and Mycoses Study Group (MSG) formed a consensus committee. They gave the first standard definition of invasive fungal infections. The definition included three categories of patients depending upon the probability of a diagnosis of invasive fungal infection, namely proven, probable and possible (Ascioglu et al., 2002). This classification was done on the basis of the presence or absence of certain host factors (patients at risk), clinical factors (signs/symptoms or radiological features consistent with disease entity) and mycological evidence. The definition included only immunocompromised patients with cancer and hematopoietic stem cell transplantation.

As our understanding of invasive fungal infections became better and newer technologies of indirect mycological evidence developed, the need for improvement of the original definition was realised. In, 2008 the revised definition was given by the EORTC/MSG Consensus Group (De Pauw et al., 2008). Instead of invasive fungal infections, the term “invasive fungal disease” (IFD) was adopted to clearly highlight the importance of the disease process caused by the infection. The revised definition retained the previous three categories of proven, probable and possible IFDs, the basis of classification remaining host, clinical and mycological evidence. Proven and probable categories were expanded to include indirect mycological evidence (tests based on fungal antigens, e.g., cryptococcal antigen assay in cerebrospinal fluid, galactomannan assay in plasma/serum, bronchoalveolar lavage or cerebrospinal fluid and Beta-D-glucan assay in serum). The possible category was revised to include only those patients at risk for fungal infection without any mycological evidence. Any patient, whether immunocompromised or immunocompetent, who developed a proven infection was regarded as a confirmed case of IFD.

Over the next few years, the shortcomings of the IFD definition given in, 2008 were noticed. To address these issues, ten working groups were formed by the European Organization for Research and Treatment of Cancer and the Mycoses Study Group Education and Research Consortium (EORTC/MSGERC), each one looking at one particular topic. These working groups gave their recommendations as the revised and updated definitions of IFDs in, 2020 (Donnelly et al., 2020). The groups looked at IFDs in paediatric population (group 1), radiological findings of invasive mould disease (group 2), galactomannan thresholds in different body fluids for invasive aspergillosis (group 3), utility of beta-D-glucan and T2candida assays (group 4), role of *Aspergillus* PCR (group 5), tissue diagnosis of IFDs (group 6), diagnostic criteria of *Pneumocystis jirovecii* pneumonia (group 7), clarification of the definitions of cryptococcosis (group 8) and endemic mycoses (group 9).

Interestingly, there was insufficient evidence to give recommendations regarding ICU patients except for the definition of “proven IFD”. According to, 2020 guidelines, the proven IFD requires direct evidence of the disease in the form of histopathologic, cytopathologic or direct microscopy of a specimen taken from a sterile site or positive blood culture or evidence from fungal polymerase chain reaction (PCR) with DNA sequencing in formalin-fixed paraffin-embedded tissue. The invasive nature of histopathology specimens leaves the ICU consultant with blood or sterile site culture positivity as the only source to confidently diagnose IFD. Thus, in the present scenario, the definition of probable and possible IFD in the ICU population remains blurred from both clinical and research perspectives. It can be easily understood that in the absence of robust diagnostic criteria, the epidemiological data, the clinical criteria and the treatment approaches for IFDs in the ICU population still need to be defined.

To solve the puzzle of diagnosing IFDs in ICU patients, a separate initiative under the banner of FUNDICU was taken to generate evidence for defining the complete spectrum of IFDs (Bassetti et al., 2019b). The results of the FUNDICU project are expected to clarify the diagnostic difficulties of IFD in ICU patients (Bassetti et al., 2021).

Among the various invasive fungal infections affecting ICU patients*, Candida* spp is the most common, accounting for 80% of the cases, followed by *Aspergillus* spp, which accounts for 0.3 to 19% of the cases depending upon the study cohort (Bassetti et al., 2017). *Aspergillus* spp and *Mucor* spp have been linked with increased mortality in patients suffering from viral pneumonia (e.g., influenza, SARS CoV2) (Waldeck et al., 2020; Hoenigl et al., 2022). Besides this, endemic mycoses like blastomycosis, coccidioidomycosis, histoplasmosis, etc., can cause serious systemic IFD even in the immunocompetent host.

The current article aims to summarise the recent development in the diagnosis and management of IFDs in non-neutropenic ICU patients with special emphasis on invasive candidiasis and invasive aspergillosis.

## Invasive candidiasis

It is the most common form of IFD occurring in non-neutropenic critically ill patients. By and large, *Candida* infections can be described as mucocutaneous candidiasis (e.g., oral thrush), locally invasive candidiasis (e.g., oesophageal candidiasis, vaginal candidiasis) and invasive candidiasis. Invasive candidiasis among non-neutropenic adults mainly manifests as candidemia and deep-seated candidiasis with or without candidemia (Guinea, 2014).

### Epidemiology and pathogenesis

*Candida* is a normal commensal of the human gut, but under certain predisposing conditions, there can be overgrowth followed by systemic infection (Neville et al., 2015). The transition from commensal to the pathogen is governed by a number of hosts and environment-related factors, which includes alteration in the gut microbiome by the use of broad-spectrum antibiotics, the breach in the integrity of the gut, host immune dysfunction, placement of the central venous catheter, surgery etc. These factors tip the balance of microbiota towards colonisation with drug-resistant organisms and overgrowth of fungal pathogens. Other mechanisms of acquiring *Candida* infection include contamination of indwelling devices with the hands of healthcare workers and formation of drug-resistant biofilms (*Candida* spp).

The epidemiology of invasive candidiasis shows significant geographical variation. Although *Candida albicans* account for 40 to 50% of cases across the globe, *non-albicans* are a frequent cause of concern due to their tendency to show azole resistance (Guinea, 2014). *Candida glabrata* (renamed as *Nakaseomyces glabrata*) is one of the most commonly isolated non-albicans and is notorious for reduced antifungal drug susceptibility (Hassan et al., 2021). Another difficult-to-treat isolate is *Candida auris*, an emerging drug-resistant pathogen responsible for several outbreaks in the past decade. It is known to survive on human skin and tough environmental conditions, facilitating rapid transmission in intensive care units (Cristina et al., 2023).

The recently conducted EUCANDICU study done across nine European countries reported 7.07 episodes of ICU-acquired invasive candidiasis per, 1000 ICU admissions with a crude 30-day mortality of around 42%. The study reported *Candida albicans* (57%) as the most common isolate, followed by *Candida glabrata* (21%) *and Candida parapsilosis* (13%) (Bassetti et al., 2019a). A study evaluating the distribution and trends of invasive candidiasis in the USA from, 2009 to, 2017 showed an overall incidence of 90 cases per 100,000 patients, with a stable trend during the study period (Ricotta et al., 2021). Similar to the EUCANDICU, *Candida albicans* (48%) was the most common isolate, followed by *Candida glabrata* (24%) *and Candida parapsilosis* (11%). A lab-based study from Asia reported *Candida albicans* as the most frequently isolated species (41.3%). Among the various non-albicans, *Candida tropicalis* (25.4%) was the most common isolate, followed by *Candida glabrata* (13.9%) and *Candida parapsilosis* (12.1%) (Tan et al., 2015).

Patients with chronic liver disease, chronic kidney disease, diabetes and those requiring -multiple blood transfusions can develop invasive candidiasis due to immune dysfunction. A recently conducted meta-analysis, including 34 studies and twenty-nine risk factors for invasive candidiasis among critically ill patients, showed the use of broad-spectrum antibiotics (OR, 5.6; 95% CI, 3.6- 8.8) as the highest predisposing factor (Thomas–Rüddel et al., 2022). Other risk factors reported in different studies are multiple-site *Candida* colonisation or single-site heavy colonisation, use of central venous catheters, surgery (more common if involves a breach in the continuity of gut), pancreatitis, prolonged pre-ICU hospitalisation etc (Lau et al., 2015; Ahmed et al., 2014).

### Diagnosis of invasive candidiasis

Among ICU patients, invasive candidiasis can present as candidemia or deep-seated candidiasis or deep-seated candidiasis with candidemia. Intra-abdominal candidiasis is the most common form of deep-seated candidiasis and frequently manifests as abdominal abscess, *Candida* peritonitis following a breach in the continuity of the gut or infection of the pancreatic necrosum in patients with pancreatitis (Vergidis et al., 2016).

*Proven invasive candidiasis;* Labelling a case as proven invasive candidiasis requires one or more of the following (Donnelly et al., 2020)

- A positive blood culture,
- A positive culture obtained from a specimen collected by a sterile procedure (including newly [<24hrs] placed drain) from a normally sterile site showing clinical or radiological features of infectious process,
- Histopathology, cytopathology or direct microscopy of a specimen collected by a sterile procedure from a normally sterile site showing budding yeast cells, which is further confirmed by culture or PCR. (Note that the presence of pseudo-hyphae or true hyphae alone is not confirmatory of *Candida* as they can be seen with other fungi like *Trichosporon* spp, *Geotrichum* spp etc.
- A positive PCR followed by DNA sequencing when yeast is detected in formalin- fixed paraffin -embedded tissue.

Blood culture sensitivity is 50 to 95% with a turnaround time of 3 to 7 days for candidemia and is even lower for deep-seated candidiasis without candidemia (Clancy and Nguyen, 2013; Tabak et al., 2018; Fernandez et al., 2009). Turnaround time can vary according to the species, as some may grow slower (e.g., *Candida glabrata*) than others.

Traditionally, identification of the fungal pathogen is made by using macroscopic and microscopic techniques, which are labour–intensive, time–consuming and largely dependent upon the expertise of the clinical mycologist. With recent technological advances, there has been considerable improvement in the utilisation and interpretation of fungal blood cultures. Regular surveillance blood cultures can be used in high–risk patients for early diagnosis. Matrix Associated Laser Desorption/Ionization Time of Flight Mass Spectrometry (MALDI–TOF MS) is a promising alternative for the rapid identification of *Candida* species and *in–vitro* antifungal susceptibility testing (Terrero–Salcedo and Powers–Fletcher, 2020). The construction of libraries, using MALDI–TOF MS, allows for improved species identification, as has been done for *C. auris* (Ceballos–Garzon et al., 2020)

*Probable and possible invasive candidiasis*, As mentioned before, the diagnostic criteria for “*Probable and possible*” invasive candidiasis in ICU patients are still awaited (Bassetti et al., 2021). However, applying risk prediction models and the use of non–culture–based techniques can be utilised to guide prophylactic and empirical therapy in specific patient populations. Over the past two to three decades, multiple risk prediction models were developed to identify ICU patients at risk of invasive candidiasis, namely, colonisation index, Ostrosky–Zeichner clinical prediction rule, *Candida* score, etc (Pittet et al., 1994; Ostrosky–Zeichner et al., 2011; León et al., 2006). These models were based on clinical as well as colonisation characteristics of the ICU patients. Most of these models show good negative predictive value (NPV) but poor positive predictive value (PPV). Thus, they are more helpful in excluding the diagnosis of invasive candidiasis (Ahmed et al., 2014). The use of risk scores can be further complemented with non–culture–based techniques to aid diagnosis and increase their positive predictive value (Posteraro et al., 2011). While using the risk scores, one must take into account the characteristics of the patient population where they were developed and their reproducibility in other hospitals, i.e., external validation (Hermsen et al., 2011; Ahmed et al., 2017).

Various non–culture–based techniques for the diagnosis of invasive candidiasis and recent advances are described below (Fang et al., 2023). They can be classified as

I. Serological tests,
II. Molecular–based methods
III. Biosensor–based methods
IV. Combined approaches using artificial intelligence

#### Serological tests

These tests employ the detection of antigens and/or antibodies in the patient’s serum or body fluid to identify the causative fungal pathogen. They offer the advantage of being significantly faster compared to fungal cultures and are non–invasive. Antibody–based testing can be unreliable in immunocompromised patients due to the high rates of false–negative results.

Commonly used serological tests for invasive candidiasis include beta–D–glucan assay, serum mannan/anti–mannan assay and serum CAGTA (*Candida albicans* germ tube antigen).

Beta–d–Glucan (BDG) is an integral part of the fungal cell wall, and its assay has been utilised as a pan–fungal screening test (Fang et al., 2023; Theel and Doern, 2013). Serum BDG levels are raised in patients infected with *Candida* spp, *Aspergillus* spp, *Pneumocystis jirovecii*, *Acremonium* spp, etc., exceptions being *Cryptococcus* spp, *Blastomycoses* and *Mucorales*. Various BDG tests are available in the market, namely, Fungitell assay, Wako –glucan test, and Goldstream Fungus (1–3)–β–D Glucan, but only Fungitell assay is FDA approved. The Fungitell assay has a sensitivity of 75 to 80% and a specificity of 60 to 80% in patients with invasive candidiasis. False positive results are seen in patients on haemodialysis, concurrent bacteraemia, immunoglobulin use, etc. The classical Fungitell assay is done in microtiter plate format with testing of 21 samples at a time, thus making it difficult to use for point–of–care testing. To overcome this difficulty, Fungitell STAT was designed as an adaptation of the classical assay. Fungitell STAT is used as a single–patient kit–based test which classifies patients as positive, negative or indeterminate (D'Ordine et al., 2021).

#### Molecular–based methods

Different types of fungal PCRs are used for rapid and accurate diagnosis. These tests are especially helpful in diagnosis of drug resistance and identification of species which are difficult to culture. A nonculture–based technique is T2 Candida which utilises magnetic resonance and molecular methods (PCR) for rapid diagnosis of candidemia (Ahmed et al., 2017). Two to four ml of whole blood from the patient is directly inserted into a fully automated T2 candida instrument which causes candida cell lysis using mechanical stress. Amplified DNA product is detected using super magnetic nanoparticles. It detects the five most commonly pathogenic *Candida* species (*C. albicans, C. tropicalis, C. parapsilosis, C. krusei, C. glabrata*) within a turnaround time of fewer than 5 hours. T2 Candida has a sensitivity of 91% and specificity of 94% for candidemia but has poor sensitivity for detecting deep–seated candidiasis without candidemia (Tang et al., 2019).

PCR–based tests for the direct detection of Candida DNA in whole blood represent excellent technology for rapid diagnosis (Avni et al., 2011). Multiplex PCR enables the detection of multiple pathogens in a single test by employing distinct pairs of primers for each target. Real–time multiplex PCR (m–PCR) like Septifast (Roche Diagnostics) is now widely used as an advance molecular assay (Fuchs et al., 2019). The technology helps in the quick detection of multiple organisms {including both bacterial (e.g*. Escherichia coli, Klebsiella pneumoniae, Acinetobacter baumanii, Pseudomonas aeruginosa, Enterococcus faecium, Staphylococcus aureus*) and fungal pathogens (e.g. *Candida albicans, Candida glabrata, Candida krusei, Candida parapsilosis, Candida tropicalis, Aspergillus fumigatus)*} using a single test. The sensitivity and specificity of m–PCR have been reported to vary between 60% to 100% and 90 to 96%, respectively, in various studies (Fang et al., 2023; Fuchs et al., 2019).

Among the various upcoming combined PCR approaches in fungal diagnostics are Sepsis Flow Chip and ePlex (Fang et al., 2023). The Sepsis Flow Chip uses multiplex PCR and reverse dot–blot hybridization to detect not only the pathogen but also their genetic resistance determinants in around 40 bacterial and fungal pathogens (Galiana et al., 2017). ePlex is an automated diagnostic platform which utilises microfluidics, PCR and electrochemical detection techniques for identification of pathogens in positive blood cultures (Tansarli and Chapin, 2022).

#### Biosensor based methods

Biosensors are a type of analytical devices which convert the biological process into measurable signals (Hussain K et al., 2020; Lorenzo–Villegas et al., 2023). Depending upon the type of signal generated the biosensors can be classified as electrochemical, optical, piezoelectric and thermometric (Fang et al., 2023). A detailed description of each is beyond the scope of this review. The use of biosensors is expected to revolutionise the point of care testing and rapid diagnosis.

#### Combined approaches using artificial intelligence

Artificial intelligence systems are typically designed to mimic human cognitive functions, and they often involve the use of algorithms, data, and self–correction (Fang et al., 2023). Machine learning is a branch of artificial intelligence which uses historical data to learn, recognize patterns, and make predictions or decisions. Currently artificial intelligence is being used to assist in interpretation of microscopic images of fungal structures and automated histopathological analysis (Singla et al., 2023).

### Management of invasive candidiasis

Treatment strategies for invasive candidiasis include prophylaxis (risk–driven), pre–emptive (colonisation or biomarker–driven), empirical (fever–driven) and targeted therapy (culture–driven). Different societies have published their guidelines for the management of invasive candidiasis (see **Table 1**), however, clinicians should tailor the therapy according to the unique host factors, local species distribution and rate of antifungal resistance (Pappas et al., 2016; Martin–Loeches et al., 2019; Keighley et al., 2021). Looking at the complexity of diagnosing invasive candidiasis, ICUs should implement antifungal stewardship programs, as, at one end, delayed initiation of antifungal therapy is associated with increased risk of death, while at another end, overuse is associated with the emergence of resistant strains (Hamdy et al., 2017).

**Table 1:** Comparison of current recommendations of international guidelines for invasive candidiasis in ICU patients.

	IDSA 2016* (Pappas et al., 2016)	ESICM/ESCMID task force 2019** (Martin–Loeches et al., 2019)	AUSTRALASIAN guidelines 2021 (Keighley et al., 2021)
Prophylaxis	• **Fluconazole** (loading 800mg {12 mg/kg} once daily maintenance 400 {6 mg/kg} once daily) in high–risk patients in adult ICUs with invasive candidiasis rates >5% • **Echinocandin** (Caspofungin, loading dose 70mg once daily then 50 mg once daily or Micafungin 100 mg once daily or Anidulafungin, loading dose 200 mg once daily followed by maintenance dose of 100 mg once daily) can be used as an alternative • **Daily chlorhexidine bath**	Not recommended	Not recommended
Pre–emptive	Not recommended	Not recommended	Not recommended
Empirical	• **Echinocandins** (Caspofungin, loading dose 70mg once daily then 50 mg once daily or Micafungin 100 mg once daily or Anidulafungin, loading dose 200 mg once daily followed by maintenance dose of 100 mg once daily) first line therapy in patients with fever and risk factors • **Fluconazole** (loading 800mg {12 mg/kg} once daily • maintenance 400 {6 mg/kg} once daily) and ** Liposomal amphotericin** B (3 to 5 mg/kg daily) are alternatives • Duration 2 weeks for suspected candidemia cases • Stop treatment after 4 to 5 days if no evidence of candidemia or no clinical response	• **Echinocandins** first line therapy for patients in septic shock and multiorgan failure with more than one extra– digestive site colonization • **Fluconazole** (weight–based dosing, loading 12 mg/kg once daily, maintenance 6 mg/kg once daily) for patients with low severity of illness • **Liposomal amphotericin B** is an alternative and preferred over other lipid formulations	• Consider in patients with septic shock and multiorgan failure with more than one extra–intestinal site colonization
Targeted (for candidemia)	• **Echinocandin** (Caspofungin, loading dose 70mg once daily then 50 mg once daily or Micafungin 100 mg once daily or Anidulafungin, loading dose 200 mg once daily followed by maintenance dose of 100 mg once daily) as initial therapy • **Fluconazole** (loading 800mg {12 mg/kg} once daily maintenance 400 {6 mg/kg} once daily) as an acceptable alternative for those who are not critically ill. Higher dose Fluconazole (12mg/kg daily) in selected cases of *Candida glabrata* infection • Transition from Echinocandin to Fluconazole after 5 to 7 days for selected cases • **Liposomal amphotericin B** (3 to 5 mg/kg daily) reasonable alternative • **Voriconazole** (loading 6mg/kg twice daily maintenance 3 mg/kg twice daily) is effective but not superior to fluconazole. Step–down oral therapy in selected cases of *Candida krusei.* • Duration **2 weeks** for isolated candidemia without metastatic complications after evidence of clearance of *Candida* and resolution of symptoms	• Choice of agent same as empirical • Duration 14 days from first negative blood culture. • For patients with inadequate source control use case by case approach. • Don’t de–escalate echinocandins if intravascular catheter or foreign body cannot be removed	• **Echinocandins** are the first line agent (Caspofungin, loading dose 70mg once daily then 50 mg once daily, *increase to 70 mg once daily in critical illness* or Micafungin 100 mg once daily, increase *to 150 mg once daily in critical illness* or Anidulafungin, loading dose 200 mg once daily followed by maintenance dose of 100 mg once daily, *increase by 50–75% in critical illness*) • **Fluconazole** {loading 800 mg (up to 12 mg/kg/day), maintenance 400–800 mg (6 to 12 mg/kg/day)} or **Liposomal amphotericin B** (3 mg/kg daily) are alternatives. ** Amphotericin B deoxycholate** 0.6 –1mg/kg daily can be used but lower grade of recommendation • **Voriconazole** (loading 6mg/kg twice daily maintenance 4 mg/kg twice daily) can be used in clinically stable but NOT RECOMMENDED in critically ill • Duration of therapy, **minimum of 2 weeks** after the first negative blood culture

Choice and duration of targeted therapy deep seated candidiasis is governed by the site of infection, feasibility of source control and bioavailability of the drug at the site of infection.

*Infectious Diseases Society of America.

** European Society of Intensive Care Medicine/European Society of Clinical Microbiology and Infectious Diseases.

## Invasive aspergillosis in ICU

Invasive aspergillosis mainly manifests with pulmonary involvement among ICU patients as invasive pulmonary aspergillosis (IPA) (Koehler et al., 2019). Risk factors in ICU include previous lung conditions or comorbidities like Chronic obstructive airway disease, bronchiectasis, decompensated liver disease, chronic heart failure, adult respiratory distress syndrome (ARDS) etc (Taccone et al., 2015). It can also manifest as rapid deterioration of respiratory function in ICU patients infected with influenza (Influenza–associated pulmonary aspergillosis, IAPA) or SARSCoV2 (Covid–associated pulmonary aspergillosis, CAPA) (Koehler et al., 2019; Lamoth, 2022).

A multicentre study which included 30 ICUs from 8 countries, found *Aspergillus fumigatus* as the most common (92%) species causing invasive aspergillosis (Taccone et al., 2015). Other species reported in the literature are *Aspergillus flavus, Aspergillus terreus, Aspergillus niger etc.* Morality due to invasive pulmonary aspergillosis varies between 65 to 90% (Blot et al., 2019). Invasive pulmonary aspergillosis is one of the most common “missed diagnoses” found in autopsy studies of ICU patients (Zhang et al., 2014).

### Diagnosis of invasive pulmonary aspergillosis

Diagnosing invasive aspergillosis in ICU is even more challenging than invasive candidiasis due to multiple factors like poor sensitivity (1–5%) of blood cultures, difficulty in performing lung biopsy in mechanically ventilated patients, difficulty in differentiating colonisation from infection in the respiratory specimen, non–specific clinical and radiological features etc.

The inability of EORTC/MSG definition (2008) to identify invasive pulmonary aspergillosis in ICU patients was realised at least a decade ago. In, 2012, Blot et al. gave *Asp* ICU algorithm to differentiate *Aspergillus* colonisation from infection of the respiratory tract. Patients qualifying the criteria were labelled as putative aspergillosis to differentiate from the classical definition of proven, probable and possible aspergillosis given by the guidelines. The algorithm required Aspergillus positive endotracheal aspirate culture as the entry point. *Asp ICU* algorithm had a sensitivity of 92% and specificity of 61%, and it outperformed the EORTC/MSG definition among ICU patients (Blot et al., 2012; Koulenti et al., 2014).

With the emergence of influenza–associate pulmonary aspergillosis (IAPA), EORTC/MSG definition, as well as *Asp* ICU criteria, did not work well. Therefore a 29–member committee of international experts gave a new case definition of influenza– associated pulmonary aspergillosis (Verweij et al., 2020). The definition required clinical features of influenza–like illness along with a temporally related positive influenza PCR or antigen as the entry criteria. The authors suggested that the consensus definition could be useful for defining covid–associated pulmonary aspergillosis (CAPA) also.

Recently, the EORTC/MSGERC ICU working group has proposed a definition of invasive pulmonary aspergillosis. The salient features are given below (Bassetti et al., 2021).

*Proven IPA*, It requires evidence of filamentous growth with the background of tissue damage.

- A positive culture obtained from a specimen collected via a sterile procedure from a normally sterile site, along with clinical/radiological abnormalities suggestive of infectious disease process (excluding bronchoalveolar lavage fluid, nasal sinuses and urine sample)
- Histopathology, cytopathology or direct microscopy of a specimen collected via a sterile procedure showing hyphae compatible with *Aspergillus* spp. along with tissue damage, which is further confirmed by culture or PCR. A positive PCR followed by DNA sequencing when mould is detected in formalin– fixed paraffin –embedded tissue.

*Probable IPA*, It requires the patient to qualify for all the following criteria

- Mycological evidence, any one of the following

 o Cytology, direct microscopy or culture showing the presence of aspergillus in lower respiratory tract specimen

 o A positive galactomannan assay (>0.5 in plasma/serum or >0.8 in bronchoalveolar lavage)

- Clinical/radiological factors,

 o Any 1 of the following four patterns on CT chest, dense well–circumscribed lesion (with or without halo sign), cavity, air crescent sign, consolidation (wedge and segmental or lobar)

 o For (Aspergillus tracheobronchitis), features of tissue damage in the form of ulcers, nodules, plaque or pseudomembrane on bronchoscopy.

- Host factors, any of the following, Glucocorticoids (prednisolone or equivalent >20 mg per day), neutropenia or neutrophil dysfunction, chronic respiratory disease, decompensated chronic liver failure, immunosuppressants, haematological malignancies, transplant patients, HIV, viral pneumonia (severe influenza, Covid–19)

Galactomannan assay is the most widely used and studied assay among the various non–culture–based techniques to diagnose invasive pulmonary aspergillosis. It is an antigen–based assay (polysaccharide cell wall component) used for testing *Aspergillus* spp infection in serum, bronchoalveolar lavage fluid (BAL) and cerebrospinal fluid (CSF). The only FDA–approved galactomannan test is Platelia Aspergillus enzyme immune assay (EIA) by Bio–Rad. Its overall sensitivity and specificity range between 67 to 100% and 86 to 100%, respectively (Fang et al., 2023). Sulahian et al. compared the Fungitell β–glucan (BG) assay with the galactomannan (GM) test in a case–control study including 105 patients of invasive aspergillosis and 147 controls. BDG Assay was found to be more sensitive (97% versus 82%, *P* = 0.0001) but less specific (81% versus 49%, *P* < 0.0001) as compared to galactomannan assay for diagnosing invasive aspergillosis (Sulahian et al., 2014).

Among molecular–based methods, various types of PCRs have been discussed in the section on invasive candidiasis. Several notable advances merit special mention in the diagnosis invasive aspergillosis.

AsperGenius is an innovative multiplex PCR that not only identifies the presence of *Aspergillus* species but also detects four resistance–associated mutations (RAMs, TR34/L98H/T289A/Y121F) in the CYP51A gene responsible for azole resistance (Fang et al., 2023). Consequently, this method offers the benefit of simultaneously identifying the causative organism and detecting potential drug resistance.

Recently, digital droplet PCR (ddPCR), a novel form of PCR, is being utilized for the detection and quantification of fungal pathogens, yielding superior results compared to quantitative PCR, especially in the detection of *Aspergillus fumigatus* and *Aspergillus flavus* in respiratory samples (Chen et al., 2021).

### Management of invasive aspergillosis

According to IDSA, 2016 guidelines, voriconazole (6 mg/kg twice daily then 4 mg/kg IV twice daily, oral 200 to 300 mg twice daily or weight–based dosing) is the drug of choice for the treatment of invasive aspergillosis (Patterson et al., 2016). Early initiation of antifungal therapy is warranted in patients with an index of clinical suspicion. Liposomal amphotericin B (3 to 5mg/kg IV) or isavuconazole (200 mg IV 8hrly for 6 doses then 200 mg once daily) can be used as alternative therapy. Combination therapy using voriconazole and echinocandin can be used in selected cases.

Echinocandins (Caspofungin {70 mg IV loading then 50 mg IV daily} or Micafungin {100 to 150 mg IV daily}) can be used as salvage therapy, but their routine use as primary agents is not recommended as they are fungistatic against moulds. Anidulafungin is not mentioned in this context due to the limited literature on its use in patients with invasive pulmonary aspergillosis. Other drugs that can be used as salvage therapy include amphotericin B lipid complex (ABCL {5mg/kg IV once daily}), posaconazole (oral tablet/IV 300 mg twice daily loading, then 300 mg once daily or oral suspension 200mg thrice daily) or Itraconazole (oral suspension 200 mg twice daily) (Patterson et al., 2016).

Recommended duration of therapy is 6 to 12 weeks depending on disease severity and efficacy of treatment and infection clearance. Therapeutic drug monitoring can help improve patient outcomes and prevent treatment failures. ICU patients often experience multiple organ dysfunction and have comorbidities that can impact the voriconazole drug levels. Voriconazole, with its nonlinear pharmacokinetics, extensive hepatic metabolism and multiple drug–drug interaction requires careful monitoring to ensure optimal therapeutic levels and effectiveness in complex medical scenarios.

Isavuconazole, the newest azole, was found to be non–inferior/equally effective to voriconazole in a recently conducted trial (SECURE) on invasive mould disease (Maertens et al., 2016). The drug is better tolerated than voriconazole and is considered a useful alternative.

## Antifungal overview

**Figure 2:**
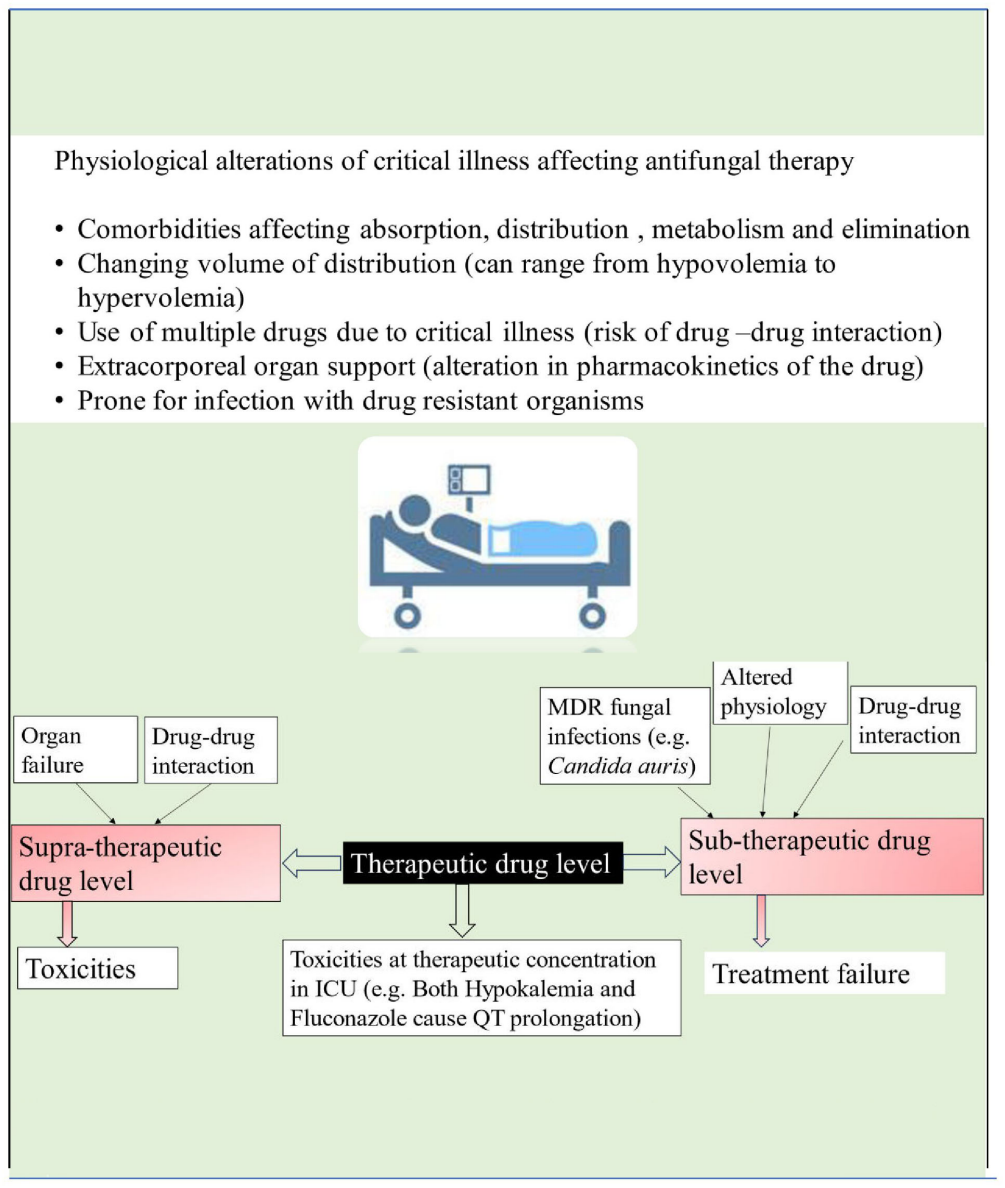
Key considerations in prescribing antifungal therapy in ICU patients.

Patients in the intensive care unit who meet the criteria for antifungal therapy are generally characterized by extreme severity of illness and presence of multiple organ dysfunction. The pathophysiological changes of critical illness cause alteration in the pharmacokinetics and pharmacodynamics of many drugs, including antifungals. These changes are attributed to gastrointestinal dysfunction causing unpredictable drug absorption, changes in fluid status, protein binding, volume of distribution, and changes in hepatic and renal function, causing altered metabolism and drug clearance (see **Figure 2**). Given these complexities, it is essential to consider individual patients’ clinical status, organ function and pharmacokinetic/pharmacodynamic alterations when prescribing antifungal medications (Dietrich et al., 2017; Abdul–Aziz et al., 2020).

There is a limited choice of antifungals available for clinical use, with four main classes currently in use: azoles, polyenes, pyrimidine analogues, and echinocandins. The pharmacokinetics/pharmacodynamics (PK/PD) parameter that best correlates with the antifungal efficacy of azoles and echinocandins is AUC _0–24_/MIC (Abdul–Aziz et al., 2020). For Amphotericin B, the pharmacokinetics/pharmacodynamics (PK/PD) parameter of interest governing antifungal efficacy is C_max_/MIC (Abdul–Aziz et al., 2020). Recently, therapeutic drug monitoring for antifungals is increasingly being used for treatment optimization and prevention of both sub and supratherapeutic drug concentrations (see **Figure 2**). According to a position paper published by a panel of experts from various international committees, routine therapeutic drug monitoring is recommended for voriconazole in critically ill patients. However, it is neither recommended nor discouraged for other azoles, echinocandins, or 5–fluorocytosine (Abdul–Aziz et al., 2020).

Azoles act by inhibiting the synthesis of ergosterol, an essential component of the fungal cell membrane. They generally exhibit fungistatic properties, except for voriconazole, which demonstrates fungicidal activity against *A. fumigatus*. The fungistatic characteristic of azoles creates significant selection pressure for the development of resistance. Additionally, some of the species of *Candida* (e.g. *C. glabrata, C. krusei*) are intrinsically “less susceptible” to this class of antifungals. However, the newer azole isavuconazole has been found to have good *in–vitro* activity against *C. glabrata and C. krusei*.

Azoles, in particular itraconazole, posaconazole and voriconazole, show poor water solubility, considerable variation in systemic dispersion and have significant adverse drug reactions. Their therapeutic drug monitoring ensures safety and prevents treatment failure (Baracaldo–Santamaría et al., 2022).

Fluconazole and isavuconazole are water–soluble compounds showing stable pharmacokinetics and do not require routine therapeutic drug monitoring. In the case of fluconazole, therapeutic drug monitoring is useful for patients suffering from CNS disease, as bioavailability in cerebrospinal fluid can vary between 50 to 90%. Other conditions requiring therapeutic drug monitoring of fluconazole include renal dysfunction due to its renal excretion and cases where organisms with high MIC cause the infection. Hepatotoxicity and gastrointestinal side effects are important concerns when using azoles. All azoles cause QT prolongation, except for isavuconazole, which causes QT shortening. These considerations are important for ICU patients who are already prone to arrhythmias.

*Polyenes*, act by binding to fungal ergosterol and destabilisation the fungal cell membrane. Amphotericin B and Nystatin are the currently available polyenes.

Amphotericin B is one of the most commonly used antifungals among ICU patients and has the broadest spectrum of activity. It is insoluble in water and therefore requires addition of an excipient to gain stability in aqueous solution. Sodium deoxycholate was initially the first excipient used to facilitate stable micellar suspensions of amphotericin B, however, this compound was associated with significant side effects (Laniado–Laborín and Cabrales–Vargas, 2009). Subsequently, safer formulations of amphotericin B were developed, including liposomes, emulsions, and lipid complexes (Hamill, 2013). Adverse effects due to amphotericin in broadly classified as those due to a) direct toxicity and b) infusion related side effects. Direct toxicity occurs due to amphotericin B having affinity for cholesterol present in mammalian cell membrane besides its binding to fungal ergosterol. It manifests as decreased renal blood flow and tubular injury leading to nephrotoxicity. Tubular injury due to amphotericin B frequently presents as polyuria with hypokalaemia and hypomagnesemia.

Infusion related side effects occur due release of proinflammatory mediators which manifests as fever, rigor, hypotension or hypertension, nausea and vomiting (Hamill, 2013). These reactions are less common with liposomal amphotericin B (L–AmB) compared to amphotericin B deoxycholate. However, Amphotericin B Colloidal Dispersion (ABCD) causes similar or more frequent infusion–related reactions compared to conventional amphotericin B deoxycholate (Hamill, 2013).

Recently, there has been development of encochleated AmB–d (C–AmB), which is a novel lipid nanocrystal for oral therapy of serious fungal infections. C–AmB is composed of a solid lipid bilayer with calcium, rolled into a spiral form where amphotericin B molecules are entrapped. The drug becomes biologically active when phagocytic cells take up the cochlea. The cochlea structure protects the drug in harsh environments like low stomach pH and enables targeted intracellular delivery into specific cells, such as macrophages and reticuloendothelial cells. This design aims to avoid toxicities associated with traditional amphotericin B formulations (Lipa–Castro et al., 2021).

Amphotericin B is a concentration dependent antifungal and concentrations four to ten times above MIC are needed for fungicidal activity. Routine therapeutic drug monitoring is not recommended for amphotericin B as the pharmacokinetics and pharmacodynamics characteristics vary with different formulations.

*Pyrimidine analogue*, Five–flucytosine is the antifungal pyrimidine analogue which acts by destabilising the fungal DNA and RNA. Routine therapeutic drug monitoring should be used for flucytosine as it can cause dose–related hepatotoxicity, bone marrow suppression and gastrointestinal disturbances.

*Echinocandins* are the unique class of antifungals with the least systemic toxicity due to their action on fungal cell walls, which is not present in human cells. Caspofungin, anidulafungin and micafungin are the three agents in this group with equal efficacy (Ylipalosaari et al., 2021). Echinocandins block beta 1–3 glucan synthase leading to decreased beta–1–3 glucan production, which is an essential component of fungal cell walls. Echinocandins are generally well tolerated and have a favourable safety profile (Szymański et al., 2022). They are administered once daily due to their pharmacokinetic properties, including long half–lives. They are potent antifungals with concentration–dependent fungicidal activity against *Candida* and fungistatic action against *Aspergillus* species. Therapeutic drug monitoring (TDM) is not routinely needed for echinocandins (Baracaldo–Santamaría et al., 2022).

Rezafungin is a newly FDA–approved echinocandin with a remarkable extended half–life of around 133 hours, which allows for less frequent administration, leading to higher plasma concentrations early in treatment (Ham et al., 2021). Its unique once–weekly dosing feature is advantageous as it reduces the necessity for regular central line access, mitigating the associated risks of central line–related infections. Thomspon 3^rd^ et al. did a pooled analysis of data from two RCTs (phase 2 STRIVE and phase 3 ReSTORE) comparing Rezafungin with Caspofungin. Rezafungin was found non–inferior to Caspofungin with early treatment benefit, due to its dosing regimen (Thompson et al., 2023).

With the increased use of antifungal agents, there is a rise in antifungal resistance. Various mechanisms of acquiring antifungal resistance include a decrease in effective drug concentration, target site modification and metabolic bypass strategies (Cowen et al., 2014). Fungi decrease the effective drug concentration by acquiring efflux pumps (ABC transporters and MFS major facilitator superfamily), overexpression of drug targets (Erg 11 upregulation by *Candida albicans* and Cyp 51A upregulation by *Aspergillus fumigatus*) or sequestration of drug within extracellular compartments (biofilm–producing strains).

## Conclusion

Invasive fungal diseases (IFDs) are traditionally diagnosed based on host factors, clinical criteria, and mycological criteria. However, ICU patients often do not meet the classical host criteria outlined in previous guidelines, making their diagnosis challenging. Invasive candidiasis and invasive aspergillosis are the most prevalent forms of IFDs in ICU settings. Their diagnostic criteria are currently evolving with the development of various nonculture–based techniques. The completion of the FUNDICU project is eagerly anticipated, as it is expected to provide revised diagnostic criteria for IFDs in ICU patients. Advances in non–culture–based methods and artificial intelligence are anticipated to revolutionize the diagnosis and treatment of IFDs in the coming years.

Recent years have also brought advancements in the antifungal armamentarium. Isavuconazole, a newer member of the azole class, has an improved safety profile compared to other azoles, with fewer concerns related to hepatotoxicity and drug interactions. Encochleated amphotericin B, a lipid nanocrystal for oral therapy, has improved drug tolerance and ease of administration for this favourite broad–spectrum antifungal. Rezafungin, a newly introduced echinocandin, requires only once–a–week administration and offers faster clearance of candidemia compared to its older echinocandin counterparts. Overall, the choice and monitoring of antifungals in the ICU are guided by individual patient factors, drug characteristics, and the potential for adverse effects.

## Author contributions

AAz: Conceptualization, Supervision, Writing – review & editing. AAh: Writing – original draft, Writing – review & editing.
